# Anti-HIV Antibody Responses and the HIV Reservoir Size during Antiretroviral Therapy

**DOI:** 10.1371/journal.pone.0160192

**Published:** 2016-08-02

**Authors:** Sulggi A. Lee, Peter Bacchetti, Nicolas Chomont, Remi Fromentin, Sharon R. Lewin, Una O’Doherty, Sarah Palmer, Douglas D. Richman, Janet D. Siliciano, Steven A. Yukl, Steven G. Deeks, Peter D. Burbelo

**Affiliations:** 1 Department of Medicine, University of California San Francisco, San Francisco, CA, United States of America; 2 CR-CHUM and Department of Microbiology, Infectious Diseases, and Immunology, Université de Montréal, Montreal, Quebec, Canada; 3 The Peter Doherty for Infection and Immunity, The University of Melbourne and Royal Melbourne Hospital, Melbourne, VIC, Australia; 4 Department of Infectious Diseases, Monash University and Alfred Hospital, Melbourne, VIC, Australia; 5 Department of Pathology and Laboratory Medicine, University of Pennsylvania, Philadelphia, PA, United States of America; 6 Centre for Virus Research, The Westmead Institute for Medical Research, The University of Sydney, Sydney, New South Wales, Australia; 7 Departments of Medicine and Pathology, University of California San Diego, La Jolla, California, United States of America, Veterans Affairs San Diego Healthcare System, San Diego, CA, United States of America; 8 Department of Medicine Johns Hopkins University School of Medicine, Baltimore, MD, United States of America; 9 Veterans Affairs San Francisco Healthcare System, San Francisco, CA, United States of America; 10 Dental Clinical Research Core, National Institute of Dental and Craniofacial Research, Bethesda, MD, United States of America; Emory University School of Medicine, UNITED STATES

## Abstract

**Background:**

A major challenge to HIV eradication strategies is the lack of an accurate measurement of the total burden of replication-competent HIV (the “reservoir”). We assessed the association of anti-HIV antibody responses and the estimated size of the reservoir during antiretroviral therapy (ART).

**Methods:**

We evaluated anti-HIV antibody profiles using luciferase immunoprecipitation systems (LIPS) assay in relation to several blood-based HIV reservoir measures: total and 2-LTR DNA (rtPCR or droplet digital PCR); integrated DNA (*Alu* PCR); unspliced RNA (rtPCR), multiply-spliced RNA (TILDA), residual plasma HIV RNA (single copy PCR), and replication-competent virus (outgrowth assay). We also assessed total HIV DNA and RNA in gut-associated lymphoid tissue (rtPCR). Spearman correlations and linear regressions were performed using log-transformed blood- or tissue-based reservoir measurements as predictors and log-transformed antibody levels as outcome variables.

**Results:**

Among 51 chronically HIV-infected ART-suppressed participants (median age = 57, nadir CD4+ count = 196 cells/mm3, ART duration = 9 years), the most statistically significant associations were between antibody responses to integrase and HIV RNA in gut-associated lymphoid tissue (1.17 fold-increase per two-fold RNA increase, P = 0.004) and between antibody responses to matrix and integrated HIV DNA in resting CD4+ T cells (0.35 fold-decrease per two-fold DNA increase, P = 0.003). However, these associations were not statistically significant after a stringent Bonferroni-adjustment of P<0.00045. Multivariate models including age and duration of ART did not markedly alter results.

**Conclusions:**

Our findings suggest that anti-HIV antibody responses may reflect the size of the HIV reservoir during chronic treated HIV disease, possibly via antigen recognition in reservoir sites. Larger, prospective studies are needed to validate the utility of antibody levels as a measure of the total body burden of HIV during treatment.

## Introduction

A challenge in evaluating the success of HIV eradication strategies is the need for accurate measurements of the total body burden of persistent HIV during effective antiretroviral therapy (ART). Most HIV resides in tissues inaccessible for routine sampling (e.g., lymph nodes, gut, and spleen). Even if accessible, there are no published methods to quantify levels of replication-competent virus in tissues [1].

Several approaches have attempted to estimate the number of latently infected cells. The gold standard method is the quantitative viral outgrowth assay (QVOA) in which cells are induced to release virus and infect new cells in culture. However, this assay is difficult, expensive, and requires fresh samples for processing. QVOA also likely represent the minimal size of the reservoir, as a substantial fraction of replication-competent viruses are not induced in this assay [2]. In addition, it is unclear how many infected cells need to be induced in order to elicit viral rebound. As an alternative approach, several PCR-based assays have measured HIV-1 nucleic acids. However, the majority of HIV DNA during ART is defective [2]. Thus, PCR-based assays over-estimate the size of the viral reservoir due to amplification of defective virus [1]. Most of the HIV reservoir also persists in lymphoid tissues, yet current blood-based measures of the reservoir do not correlate well with tissue-based measures [1].

We previously showed that a highly sensitive antibody assay could distinguish HIV-infected individuals with different sizes of the viral reservoir [3]. We observed lower anti-HIV antibody levels in “the Berlin patient” (an HIV-infected patient with sustained viral suppression after a CCR5-Delta32 homozygous stem cell transplantation) compared to HIV controllers (patients maintaining viral suppression without ART). Antibody levels were also statistically significantly lower in HIV controllers compared to HIV non-controllers (whether ART suppressed or not) and higher in the Berlin patient compared to “background” levels in HIV-uninfected controls [3].

Identifying a blood-based measure that reflects the HIV reservoir size could be useful in monitoring the success of HIV eradication interventions, or even as a future clinical assay. As circulating anti-HIV antibody levels likely reflect in part, the total body antigenic burden, plasma sampling of antibodies could potentially quantify changes in tissue reservoir size over time. Here, we performed a cross-sectional analysis of anti-HIV antibody responses in relation to previously collected blood and tissue measures of the HIV reservoir during long-term ART.

## Methods

Given previous data demonstrating blunted HIV-1 specific antibody responses in participants who initiate ART during acute/early infection [4–6], for this study, we only included participants from the University of California San Francisco SCOPE cohort who initiated ART during chronic (>2 years from the estimated date of HIV-1 diagnosis) to avoid potential confounding by timing or ART initiation. Additional inclusion criteria were confirmed HIV-1 diagnosis, plasma HIV RNA <40 copies/mL for ≥3 years, and CD4+ T cell counts >350 cells/mm^3^ during the preceding 6 months. Exclusion criteria were hospitalization, infection requiring antibiotics, vaccination, or exposure to immunomodulatory drugs in the preceding six months. The research was approved by the University of California Institutional Review Board, and all participants provided written informed consent.

The luciferase immunoprecipitation systems (LIPS) technology, employing light-emitting *Renilla* luciferase fusion proteins, was performed to measure antibodies against gp41, reverse transcriptase, integrase, protease, matrix, and capsid HIV-1 antigens [3]. Since a previous *Renilla* luciferase-gp120 C-terminal antigen fusion exhibited poor diagnostic performance in HIV-infected individuals [3], a new N-terminal fusion was employed using a mammalian expression vector, pGaus3, containing *Gaussia* luciferase without its start methionine. PCR was used to amplify the coding sequence of gp120 cDNA, and the resulting restriction fragment was subcloned into the pGaus3 vector. Details of its construction are available upon request. Samples were tested for antibody responses, blinded to HIV reservoir status.

Fourteen previously collected HIV reservoir measures from peripheral blood samples were included: total and 2-LTR DNA by rtPCR [7] or droplet digital PCR [8], integrated DNA by *Alu*-*LTR* PCR [7] or *Alu*-*gag* PCR [9], cell-associated unspliced (CA-US) RNA by rtPCR [10], multiply-spliced RNA by TILDA [11], residual plasma HIV RNA by single copy assay [12], and replication-competent virus by QVOA [2]. In addition, two HIV reservoir measures from gut-associated lymphoid tissue (GALT)—total HIV DNA and RNA by rtPCR [1]—were included in this study.

We fit linear regression models to estimate the fold-change in HIV antibody levels per two-fold change in each HIV reservoir measure. Multivariate models were adjusted for age and duration of ART suppression. We did not include nadir or proximal CD4+ T cell count or pre-ART HIV RNA, as these factors could be intermediates or colliders in a potential causal pathway between HIV reservoir size and anti-HIV antibody responses. A Bonferroni-adjustment of P<0.05 for a total of 112 associations produced a bound of P<0.00045. Spearman correlations were also performed to assess univariate associations. Statistical analyses were performed using STATA version 14 (StataCorp).

## Results

The 51 chronically HIV-infected participants were mostly male (96%) and had a median age of 57 years, pre-ART HIV RNA of 4.7 log_10_copies/mL, nadir CD4+ T cell count of 196 cells/mm^3^, and proximal CD4+ T cell count of 688 cells/mm^3^ (Table 1). The median years from HIV diagnosis to ART initiation was 6, and the median duration of ART suppression was 9 years.

**Table 1:** Characteristics of HIV-infected antiretroviral therapy (ART)-suppressed study participants.

	N = 51
Sex (male)	49 (96%)^a^
Age (years)	57 (50–62)^b^
Pre-ART HIV RNA (log_10 (_copies/mL)	4.7 (4.1–5.1)
Nadir CD4+ T cell count (cells/mm^3^)	196 (120–270)
Time from HIV infection to ART initiation (years)^c^	6 (4–11)
Duration of ART (years)	9 (6–13)
Proximal CD4+ T cell count (cells/mm^3^)^d^	688 (539–841)

^a^ Frequency and percent.

^b^ Median and interquartile range.

^c^ Estimated number of years from time from HIV infection to ART initiation by self-report.

^d^ “Proximal” refers to most recent CD4+ T cell count in relation to HIV reservoir and antibody measurements.

**Fig 1:**
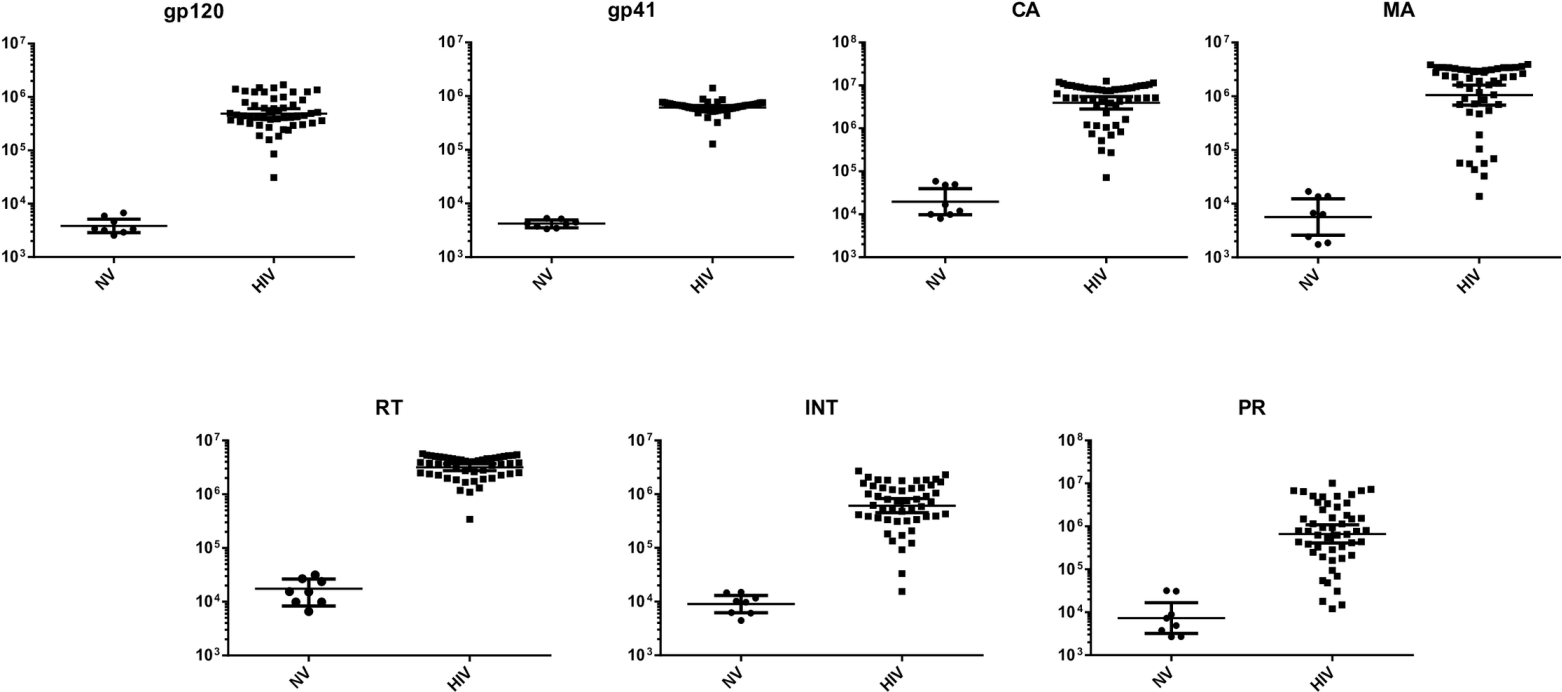
Antibody profiles against seven different HIV-1 antigens in the HIV cohort used for reservoir measurements. The antibody levels against each of the seven HIV proteins were determined in HIV-uninfected (N = 8) and HIV-infected ART-suppressed (N = 51) participants. The antibody levels are plotted on the Y-axis using a log_10_scale, and the geometric mean with 95% CI are shown. Comparison between the two groups for all the antigens revealed highly statistically significant higher antibody responses in HIV-infected participants (p<0.0001), as expected. Abbreviations: GP120 = envelope glycoprotein 120; GP41 = envelope glycoprotein 41; RT = reverse transcriptase; INT = integrase; PR = protease; MA = matrix; CA = capsid; NV = HIV-uninfected; HIV = HIV-infected.

LIPS antibody profiling detected robust seropositive responses in all samples against all seven HIV-1 antigens when compared to the Berlin patient and to HIV-uninfected controls (Fig 1). Statistical testing with the Mann Whitney U test revealed that the differences in antibody levels between the HIV-uninfected and HIV-infected participants were all highly significant (P<0.0001). Humoral responses to the HIV-1 Pol antigens (reverse transcriptase, integrase, and protease) were moderately correlated with each other (R = 0.52–0.68, P≤0.001) (S1 Fig), and antibody responses to HIV-1 Gag antigens (matrix and capsid) were correlated with each other (R = 0.62, P<0.001). Antibody responses to HIV-1 Env antigens (gp120 and gp41) were minimally correlated with each other (R = 0.17, P = 0.22). HIV reservoir measures were highly correlated with each other, though several of these correlations were based on few observations (S2 Fig).

Linear regressions using log-transformed reservoir measurements as predictors and log-transformed antibody as outcome variables demonstrated that the most statistically significant associations, assessed by lowest p-values, were between integrated HIV DNA in resting CD4+ T cells (by *Alu-gag* PCR) and antibody responses to matrix (Table 2) and between HIV RNA in GALT and integrase antibody levels (Table 3). There was a 0.35-fold decrease in matrix antibody levels per two-fold increase in integrated HIV DNA in resting CD4+ T cells (P = 0.003) and a 1.17-fold increase in integrase antibody levels per two-fold increase in HIV RNA in GALT (P = 0.004). However, neither of these associations met strict Bonferroni-adjusted criteria.

**Table 2:** Estimated effects of measures of peripheral blood total or integrated HIV-1 DNA on anti-HIV antibody responses^a^.

**Total HIV-1 DNA**						
	**rtPCR (CD4)**		**ddPCR (PBMC)**		**ddPCR (rCD4)**	
	**(N = 46)**		**(N = 20)**		**(N = 11)**	
**Antibody**	**Fold-change^b^**	**P^c^**	**Fold-change**	**P**	**Fold-change**	**P**
**GP120**	1.02 (0.92, 1.12)	0.75	1.06 (0.93, 1.2)	0.35	1.08 (0.91, 1.29)	0.35
**GP41**	1.00 (0.96, 1.03)	0.88	1.02 (0.99, 1.06)	0.16	0.99 (0.93, 1.04)	0.57
**RT**	0.99 (0.93, 1.06)	0.84	1.05 (0.98, 1.12)	0.13	1.01 (0.92, 1.10)	0.89
**INT**	1.05 (0.92, 1.19)	0.47	1.16 (0.98, 1.37)	0.074	1.14 (0.96, 1.34)	0.12
**PR**	0.94 (0.78, 1.14)	0.51	1.37 (0.97, 1.95)	0.070	1.28 (0.85, 1.93)	0.21
**MA**	0.95 (0.79, 1.14)	0.60	0.90 (0.68, 1.19)	0.46	0.92 (0.68, 1.25)	0.56
**CA**	0.92 (0.81, 1.04)	0.18	0.89 (0.67, 1.19)	0.42	0.84 (0.63, 1.12)	0.20
**Integrated HIV-1DNA**						
	***Alu*-*LTR* PCR (CD4)**		***Alu*-*LTR* PCR (PBMC)**		***Alu-gag* PCR (rCD4)**	
	**(N = 46)**		**(N = 12)**		**(N = 10)**	
**Antibody**	**Fold-change^b^**	**P^c^**	**Fold-change**	**P**	**Fold-change**	**P**
**GP120**	1.07 (0.96, 1.19)	0.24	1.03 (0.78, 1.36)	0.83	0.88 (0.59, 1.30)	0.47
**GP41**	1.00 (0.96, 1.04)	0.98	1.00 (0.95, 1.06)	0.97	0.98 (0.91, 1.05)	0.55
**RT**	1.04 (0.97, 1.12)	0.23	1.04 (0.90, 1.20)	0.52	0.98 (0.79, 1.20)	0.79
**INT**	1.02 (0.88, 1.18)	0.83	0.98 (0.66, 1.46)	0.90	0.72 (0.40, 1.32)	0.25
**PR**	1.19 (0.95, 1.47)	0.12	1.46 (0.63, 3.40)	0.34	0.89 (0.24, 3.29)	0.84
**MA**	1.11 (0.89, 1.37)	0.34	0.68 (0.39, 1.19)	0.16	0.35 (0.20, 0.64)	0.003
**CA**	1.03 (0.88, 1.20)	0.70	0.77 (0.45, 1.32)	0.30	0.49 (0.24, 1.01)	0.053

Abbreviations: GP120 = envelope glycoprotein 120; GP41 = envelope glycoprotein 41; RT = reverse transcriptase; INT = integrase; PR = protease; MA = matrix; CA = capsid; rtPCR = reverse transcriptase polymerase chain reaction (PCR); LTR = long terminal repeat; Gag = HIV-1 Gag protein; ddPCR = droplet digital PCR; *Alu* PCR = PCR using a primer in an *Alu* element to detect integrated HIV-1 DNA; rCD4 = resting CD4+ T cells; PBMC = peripheral blood mononuclear cells. Associations with P<0.05 are highlighted in bold font.

^a^ Linear regressions of log_2_anti-HIV antibody levels. Note: outcome measures are shown in the rows in the left column while predictor variables are shown in columns.

^b^ Fold-change in anti-HIV antibody responses per two fold-change in the HIV reservoir measure.

^c^ Bonferroni-adjusted significance cutoff would be P < 0.00045, after adjustment for 112 assessments of association.

**Table 3:** Association between measures of cell-associated HIV-1 DNA and RNA from gut-associated lymphoid tissue (GALT) and anti-HIV antibody responses^a^.

HIV-1 DNA AND RNA IN TISSUE						
	Total HIV-1 DNA		Total HIV-1 RNA^d^		HIV-1 RNA/DNA	
	rtPCR (CD4)		rtPCR (CD4)		rtPCR (CD4)	
	(N = 16)		(N = 15)		(N = 15)	
Antibody	Fold-change^b^	P^c^	Fold-change	P	Fold Change	P
**GP120**	**1.22 (1.04, 1.44)**	**0.019**	**1.12 (1.01, 1.23)**	**0.028**	1.72 (0.84, 3.54)	0.13
**GP41**	1.00 (0.95, 1.06)	0.95	1.00 (0.98, 1.03)	0.73	1.08 (0.9, 1.30)	0.39
**RT**	1.03 (0.93, 1.15)	0.49	1.02 (0.96, 1.08)	0.58	1.15 (0.78, 1.71)	0.44
**INT**	1.24 (0.99, 1.56)	0.060	**1.17 (1.06, 1.28)**	**0.004**	**2.33 (1.13, 4.83)**	**0.026**
**PR**	**1.65 (1.04, 2.63)**	**0.036**	1.29 (0.98, 1.70)	0.068	4.04 (0.58, 28.0)	0.14
**MA**	0.95 (0.66, 1.36)	0.75	1.01 (0.81, 1.25)	0.94	2.37 (0.63, 8.87)	0.18
**CA**	0.90 (0.64, 1.27)	0.52	0.94 (0.77, 1.15)	0.53	0.80 (0.21, 3.08)	0.72

Abbreviations: GP120 = envelope glycoprotein 120; GP41 = envelope glycoprotein 41; RT = reverse transcriptase; INT = integrase; PR = protease; MA = matrix; CA = capsid; rtPCR = reverse transcriptase polymerase chain reaction (PCR). Associations with P<0.05 are highlighted in bold font.

^a^ Linear regressions of log_2_anti-HIV antibody levels. Note: outcome measures are shown in the rows in the left column while predictor variables are shown in columns.

^b^ Fold-change in anti-HIV antibody responses per fold-change in the HIV reservoir measure.

^c^ Bonferroni-adjusted significance cutoff would be P < 0.00045, after adjustment for 112 assessments of association.

^d^ Normalized to levels of glyceraldehyde phosphate dehydrogenase (GAPDH), as determined by a separate rtPCR.

We also observed additional associations meeting statistical significance at P<0.05, but not at the Bonferroni-adjusted threshold -e.g., CA-US RNA in peripheral CD4+ T cells and gp120 antibody levels (fold-change 1.26, P = 0.011) (Table 4) and several comparisons between GALT HIV RNA and DNA measures and gp120 or protease antibody levels (Table 2). There was a 1.22-fold increase in gp120 antibody levels per two-fold increase in GALT HIV DNA (P = 0.019) and a 1.12-fold increase in gp120 antibody levels per two-fold increase in GALT HIV RNA (P = 0.028) (Table 2). We also observed a 1.65-fold increase in protease antibody levels per two-fold increase in GALT HIV DNA (P = 0.036). Of note, we did not observe any statistically significant associations between QVOA and anti-HIV antibody measures (Table 5).

**Table 4:** Association between peripheral blood HIV-1 DNA or RNA measures of transcription and anti-HIV antibody responses^a^.

** 2-LTR HIV-1 DNA**						
	**rtPCR (CD4)**		**ddPCR (PBMC)**		**ddPCR (rCD4)**	
	**(N = 46)**		**(N = 20)**		**(N = 11)**	
**Antibody**	**Fold-change^b^**	**P^c^**	**Fold-change**	**P**	**Fold-change**	**P**
**GP120**	1.07 (0.95, 1.19)	0.25	1.1 (0.91, 1.34)	0.28	1.13 (0.83, 1.54)	0.39
**GP41**	1.00 (0.96, 1.04)	0.97	1.04 (0.99, 1.10)	0.10	1.00 (0.91, 1.09)	0.92
**RT**	1.04 (0.97, 1.12)	0.25	1.07 (0.97, 1.18)	0.20	0.99 (0.84, 1.16)	0.87
**INT**	1.02 (0.88, 1.18)	0.84	1.15 (0.89, 1.50)	0.27	1.18 (0.87, 1.59)	0.26
**PR**	1.18 (0.95, 1.47)	0.14	1.48 (0.87, 2.54)	0.14	1.24 (0.58, 2.64)	0.53
**MA**	1.11 (0.89, 1.37)	0.35	0.88 (0.58, 1.34)	0.53	1.01 (0.59, 1.73)	0.97
**CA**	1.03 (0.88, 1.20)	0.71	1.02 (0.66, 1.59)	0.92	0.75 (0.46, 1.25)	0.24
**HIV-1 RNA**						
	**CA-US RNA**		**Plasma RNA**		**TILDA**	
	**rtPCR (CD4)**		**rtPCR (plasma)**		**msRNA (CD4)**	
	**(N = 42)**		**(N = 20)**		**(N = 18)**	
**Antibody**	**Fold-change^b^**	**P^c^**	**Fold-change**	**P**	**Fold-change**	**P**
**GP120**	**1.26 (1.06, 1.51)**	**0.011**	0.89 (0.48, 1.66)	0.71	1.09 (0.86, 1.38)	0.47
**GP41**	1.04 (0.97, 1.13)	0.28	0.90 (0.76, 1.06)	0.20	1.00 (0.94, 1.06)	0.90
**RT**	1.04 (0.92, 1.18)	0.49	0.95 (0.68, 1.32)	0.75	0.99 (0.87, 1.13)	0.92
**INT**	1.13 (0.87, 1.46)	0.35	0.92 (0.38, 2.18)	0.83	1.12 (0.80, 1.58)	0.49
**PR**	1.07 (0.73, 1.58)	0.72	1.37 (0.22, 8.48)	0.72	1.30 (0.62, 2.71)	0.46
**MA**	1.06 (0.73, 1.55)	0.75	1.24 (0.32, 4.79)	0.74	0.74 (0.43, 1.26)	0.25
**CA**	1.08 (0.82, 1.41)	0.58	1.52 (0.38, 6.09)	0.53	0.78 (0.45, 1.36)	0.36

Abbreviations: GP120 = envelope glycoprotein 120; GP41 = envelope glycoprotein 41; RT = reverse transcriptase; INT = integrase; PR = protease; MA = matrix; CA = capsid; 2-LTR = 2-long terminal repeat circles; CA-US RNA = cell-associated unspliced HIV-1 RNA; plasma RNA = plasma HIV-1 RNA by single copy assay; TILDA = tat/rev inducible multiply spliced HIV-1 RNA (msRNA) assay. Associations with P<0.05 are highlighted in bold font.

^a^ Linear regressions of log_2_anti-HIV antibody levels. Note: outcome measures are shown in the rows in the left column while predictor variables are shown in columns.

^b^ Fold-change in anti-HIV antibody responses per two fold-change in the HIV reservoir measure.

^c^ Bonferroni-adjusted significance cutoff would be P < 0.00045, after adjustment for 112 assessments of association.

**Table 5:** Association between peripheral blood measure of inducible virus and anti-HIV antibody responses^a^.

HIV-1 INFECTIOUS UNITS		
	QVOA	
	IUPM (rCD4)	
	(N = 20)	
Antibody	Fold-change^b^	P^c^
**GP120**	1.19 (0.89, 1.59)	0.22
**GP41**	0.98 (0.90, 1.07)	0.68
**RT**	1.03 (0.88, 1.21)	0.71
**INT**	0.96 (0.63, 1.46)	0.83
**PR**	1.60 (0.68, 3.75)	0.27
**MA**	1.11 (0.58, 2.14)	0.73
**CA**	0.79 (0.40, 1.53)	0.46

Abbreviations: GP120 = envelope glycoprotein 120; GP41 = envelope glycoprotein 41; RT = reverse transcriptase; INT = integrase; PR = protease; MA = matrix; CA = capsid; QVOA = quantitative viral outgrowth assay; IUPM = infectious units per million cells; rCD4 = resting CD4+ T cells. Associations with P<0.05 are highlighted in bold font.

^a^ Linear regressions of log_2_anti-HIV antibody levels. Note: outcome measures are shown in the rows in the left column while predictor variable is shown in the column.

^b^ Fold-change in anti-HIV antibody responses per two fold-change in the HIV reservoir measure.

^c^ Bonferroni-adjusted significance cutoff would be P < 0.00045, after adjustment for 112 assessments of association.

**Fig 2:**
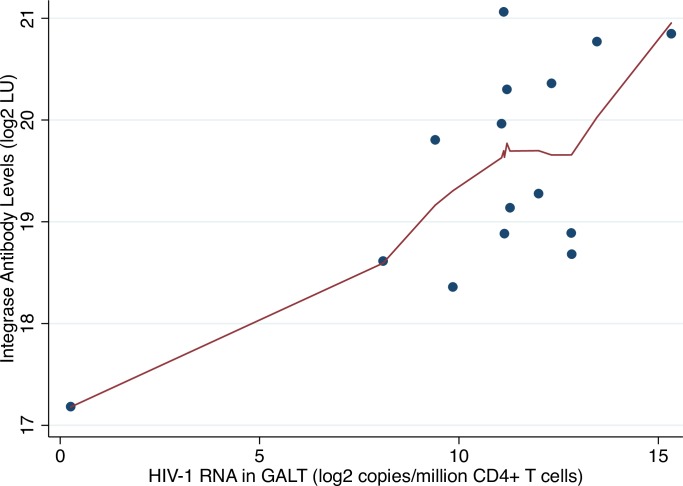
Lowess plot of log_2_-transformed HIV RNA in GALT (rtPCR) versus log_2_-transformed integrase antibody levels. Abbreviations: LU = light units; GALT = gut-associated lymphoid tissue.

**Fig 3:**
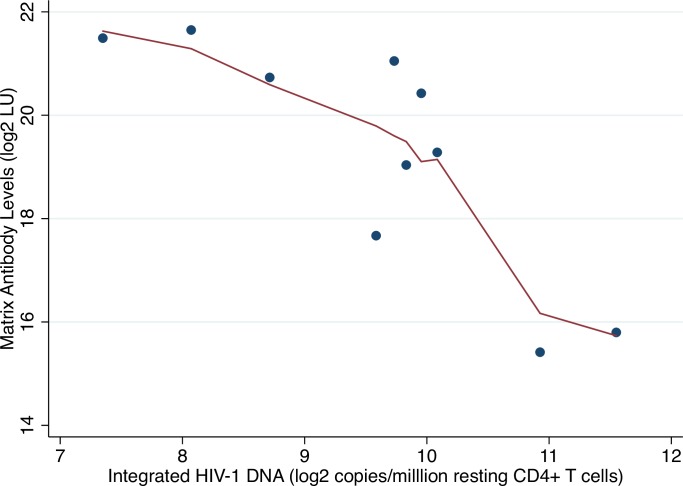
Lowess plot of log_2_-transformed integrated HIV DNA in peripheral resting CD4+ T cells (*Alu-gag* PCR) versus log_2_-transformed matrix antibody levels. Abbreviations: LU = light units.

Lowess plots revealed a potential outlier in the association between antibodies to gp120 and HIV RNA in GALT (Fig 2) but not in the association between antibodies to matrix and integrated HIV DNA in resting CD4+ T cells (Fig 3). Sensitivity analyses excluding this potential outlier did not substantially alter results (1.19 fold-increase in antibodies to integrase per two-fold increase in RNA, P = 0.002). Multivariate models adjusted for age and duration of ART did not markedly alter results (S1 and S2 Tables).

**Fig 4:**
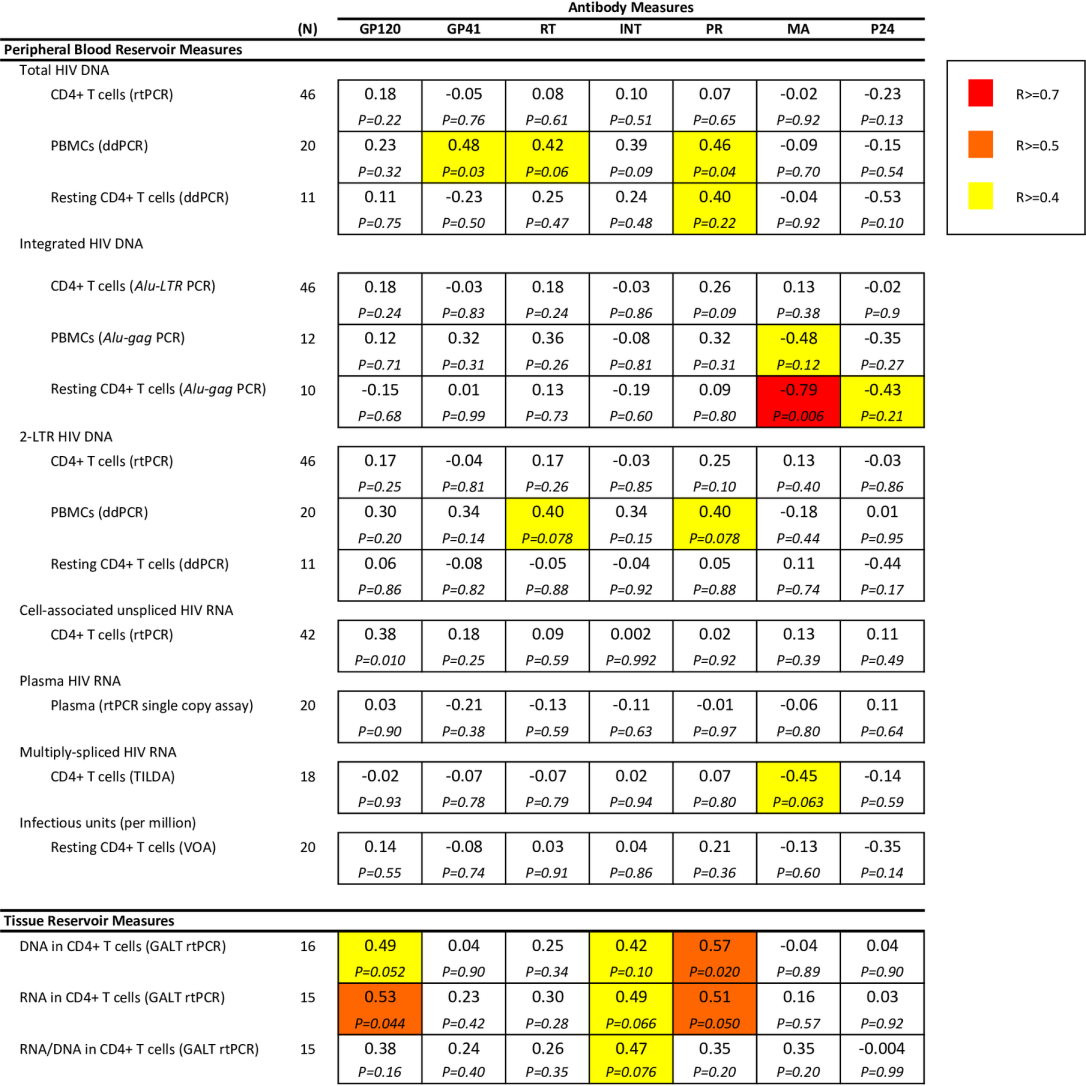
Spearman rank correlations of log_2_-transformed predictors (HIV reservoir measures in rows) versus log_2_-transformed outcomes (HIV antibody levels in columns). Correlation coefficients shown in each box with p-values below. Abbreviations: GP120 = envelope glycoprotein 120; GP41 = envelope glycoprotein 41; RT = reverse transcriptase; INT = integrase; PR = protease; MA = matrix; CA = capsid; rtPCR = reverse transcriptase polymerase chain reaction (PCR); ddPCR = droplet digital PCR; *Alu* PCR = PCR using a primer in an *Alu* element to detect integrated HIV-1 DNA; LTR = long terminal repeat; Gag = HIV-1 Gag protein; rCD4 = resting CD4+ T cells; PBMC = peripheral blood mononuclear cells; SCA = single copy assay.

We also performed Spearman rank correlations of HIV reservoir measures and anti-HIV antibody levels to confirm these associations without reliance on regression model assumptions (Fig 4). Results were similar, demonstrating the strongest correlations with integrated HIV DNA in resting CD4+ T cells and the matrix antibody levels (R = -0.79, P = 0.006), as well as between tissue reservoir measures and antibody responses to Pol (integrase, protease) and Env (gp120) antigens. HIV DNA in GALT was again associated with protease (R = 0.57, P = 0.020), gp120 (R = 0.49, P = 0.052), but also integrase (R = 0.42, P = 0.10) antibody levels, and HIV RNA in GALT was associated with integrase (R = 0.49, P = 0.066), gp120 (R = 0.53, P = 0.044), and protease (R = 0.51, P = 0.050) antibody levels.

## Discussion

There are currently no published methods to accurately and accessibly quantify levels of replication-competent virus, especially in lymphoid tissue where the majority of the HIV reservoir persists. In this study, we attempted to evaluate how well humoral responses to HIV-1 antigens reflect the size of the quantifiable HIV reservoir using a rapid and inexpensive plasma antibody assay. We observed several suggestive associations between anti-HIV antibody responses and PCR-based assays of the HIV reservoir, and intriguingly, with tissue-based RNA measures of the viral reservoir.

The suggestive associations between antibody responses and tissue-based reservoir measures were primarily with Pol proteins (integrase and protease). These results might suggest that antibodies responses against enzymes involved in HIV replication might be a better surrogate of the HIV reservoir than antibody responses against structural proteins such as gp120. Recent HIV integration site analyses emphasize the key role of enzymes involved in HIV replication in maintaining HIV latency [13, 14]. But it is unclear whether the host can generate a humoral response against non-surface HIV antigens or non-peptide (e.g., viral RNA) antigen [15]. We did observe suggestive associations between HIV RNA and DNA levels in GALT with gp120 antibody responses (Table 3), as well as between CA-US RNA in CD4+ T cells with gp120 antibody responses (Table 4). Thus another possibility is that the limited amino acid diversity of Pol sequences compared to other HIV-1 protein sequences (http://www.hiv.lanl.gov)—i.e., the more conserved protein sequences of integrase or protease—might have facilitated greater binding to HIV-1 antibodies included in the LIPS assay, and antibody binding with the more diverse Env protein sequences may have been missed.

We also found a notable negative trend with integrated HIV DNA in resting CD4+ T cells and antibody responses to Gag. Higher integrated HIV DNA levels were associated with lower antibody responses to Gag proteins (matrix and capsid), with a trend seen with the other HIV-1 antigens (Table 2). Previous studies suggest that HIV Gag+ resting CD4+ T cells are susceptible to cytotoxic T cell-mediated clearance, and the magnitude of this clearance was associated with a smaller *in vivo* HIV reservoir size [16]. In SIVmac239-infected rhesus macaques, multiple Gag-specific, but not Env-specific, CD8+ T cell responses have been associated with lower viremia, and these Gag-specific responses appeared early (approximately 2 hours post-infection, before viral integration and protein synthesis) [17]. Thus, patients who more successfully clear Gag-expressing cells might exhibit stronger antibody responses against these HIV-1 antigens. Of note, we did not observe a similar trend with integrated HIV DNA using a second *Alu-LTR*-based assay (Table 2) [7]. This discrepancy may be due to the fact that the *Alu-LTR* assay detects all proviruses including those that are deleted or hypermutated in *gag*, while the *Alu-gag* assay only measures integrated virus containing *gag* (and thus, selectively measures proviruses capable of being cleared based on Gag expression).

This study has several limitations. First, these data are cross-sectional so we cannot assess how well the observed associations persist over time, nor rule out an alternate hypothesis—that strong anti-HIV antibody responses shape the HIV reservoir size. A recent study found that HIV-infected individuals who initiated ART during acute/early infection resumed developing HIV-specific antibody responses—specifically, antibody binding diversity and functional neutralizing capacity–after analytic treatment interruption (ATI) [6]. In the study, the investigators noted that viral rebound preceded changes in anti-HIV antibody responses at 4 weeks post-ATI, concluding that anti-HIV antibody responses may not be useful predictors of time to viral rebound. However, the study only included participants who initiated ART during early HIV infection, potentially making it more difficult to evaluate the relationship between HIV persistence and antibody responses at “steady state” (early-treated individuals have both a smaller HIV reservoir size and blunted antibody responses) and the response post-ATI likely reflects a scenario more similar to initial acute HIV infection rather than the anti-HIV antibody responses during chronic treated HIV disease. The study also assessed antibody responses using an ELISA assay, which assess linear but not conformational epitopes, and performed ATI after administration of a DNA vaccine (though reportedly the intervention did not alter HIV viral load or immunogenicity).

An additional limitation of our study was the restriction to previously collected HIV reservoir data performed on a small number of participants. Several of the most statistically significant associations were observed in very few participants. We performed sensitivity analyses to evaluate potential outliers and found no evidence of influential observations. The one participant with very low HIV RNA levels in GALT (Fig 4) had similarly low reservoir size by multiple other measures. Our study was also limited by a general issue related to analyzing HIV reservoir data–the marked variability of dynamic ranges for different HIV reservoir assays. For example, accurate quantification of plasma HIV RNA or infectious virions is challenging given the very low number of copies/virions detected by these assays among ART-suppressed participants. In our small study, we did not observe a statistically significant association between residual plasma HIV RNA or QVOA with anti-HIV antibody responses. Finally, we employed a conservative Bonferroni-adjustment, which is likely overly stringent given that several anti-HIV antibody responses (Fig 2) and HIV reservoir measures were strongly correlated (Fig 3). However, this method allowed us to grossly compare results given the disparate nature of the reservoir data.

Data from other chronic viral infections suggest that long-lived immunity against viral antigens can persist and decay slowly for decades [18]. We cannot rule out the possibility that anti-HIV antibody responses are maintained via homeostatic or other mechanisms in the absence of continued antigen exposure [19] and that anti-HIV antibody responses may also be unable differentiate between defective versus replication-competent virus [2]. However, it is also yet unclear whether the HIV reservoir is completely “quiescent” [20]; there may be intermittent release of virus or cell-associated RNA from persistently infected cells. If so, one might be able to leverage the host’s ongoing, active immune response to the virus to provide a “summary” of total body persistent HIV. Thus, harnessing the humoral response may be particularly useful in quantifying changes in the size of the tissue reservoir over time; T follicular helper cells are found in lymphoid tissue and influence the proliferation, survival, and plasma cell differentiation of B cells [21]. Further studies are needed to evaluate whether anti-HIV antibody levels at “steady state” can be used to monitor long-term changes in the size of the HIV reservoir, ideally with the collection of prospective blood and tissue reservoir measures and before and after HIV latency reversal and/or analytic treatment interruption.

## Supporting Information

S1 Fig Spearman rank correlations of log_2_-transformed HIV antibody levels in N = 51 participants. Abbreviations: GP120 = envelope glycoprotein 120; GP41 = envelope glycoprotein 41; RT = reverse transcriptase; INT = integrase; PR = protease; MA = matrix; CA = capsid. Correlation coefficients shown in each box with p values below.
(TIF)

S2 Fig Spearman rank correlations of log_2_-transformed HIV reservoir measures. Abbreviations: rtPCR = reverse transcriptase polymerase chain reaction (PCR); ddPCR = droplet digital PCR; *Alu* PCR = PCR using a primer in an *Alu* element to detect integrated HIV-1 DNA; rCD4 = resting CD4+ T cells; PBMC = peripheral blood mononuclear cells; SCA = single copy assay. Correlation coefficients shown in each box with p values below.
(TIF)

S1 Table Age-adjusted linear regressions of measures of the HIV reservoir and anti-HIV antibody responses. (DOCX)

S2 Table Duration of ART-adjusted linear regressions of measures of the HIV reservoir and anti-HIV antibody responses. (DOCX)
