# Supplementary figures and images for: Anti-HIV Antibody Responses and the HIV Reservoir Size during Antiretroviral Therapy
Source: PLoS One. 2016 Aug 2;11(8):e0160192. doi: 10.1371/journal.pone.0160192 (PMC4970722; doi:10.1371/journal.pone.0160192)

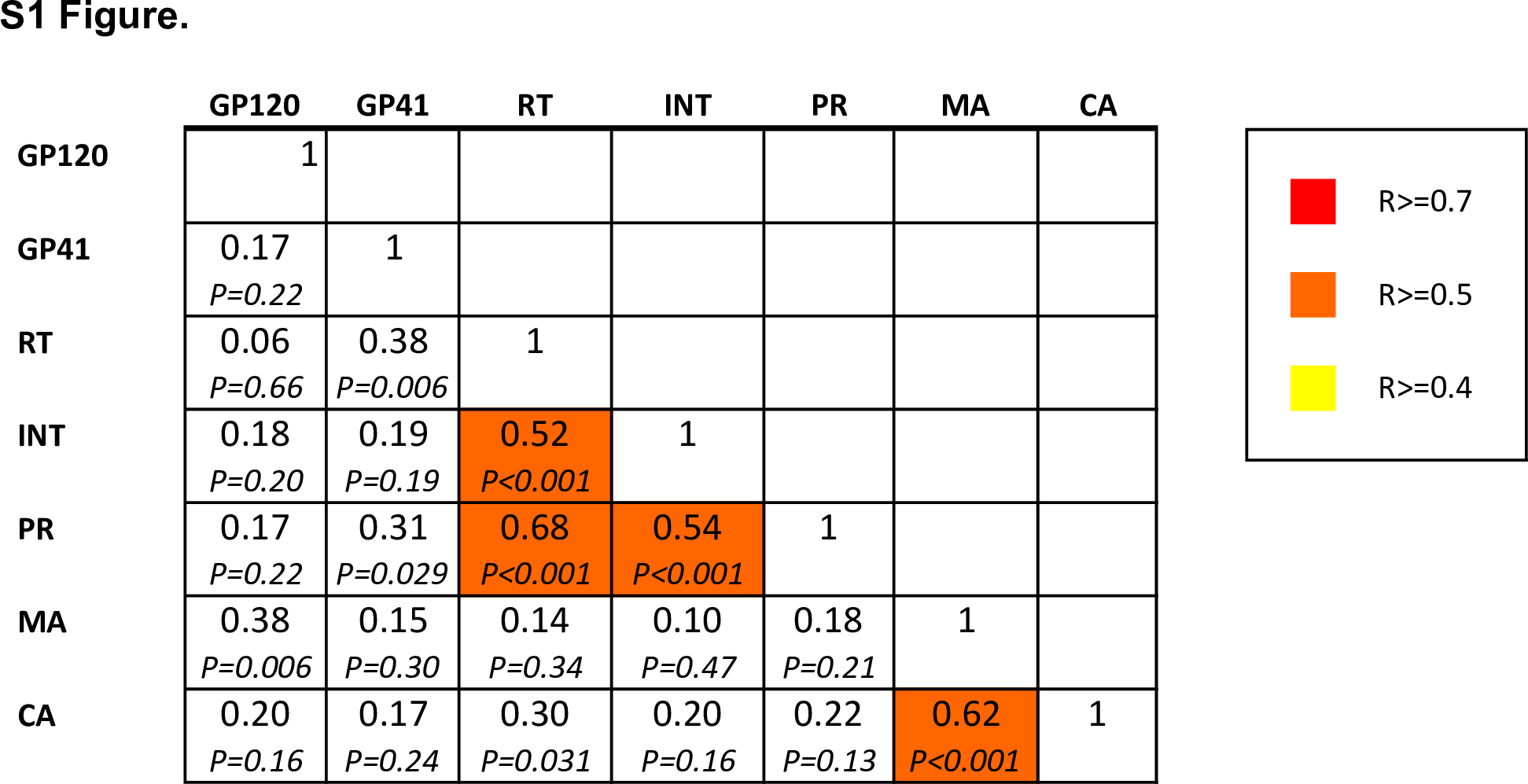

Supplement: S1 Fig — Abbreviations: GP120 = envelope glycoprotein 120; GP41 = envelope glycoprotein 41; RT = reverse transcriptase; INT = integrase; PR = protease; MA = matrix; CA = capsid. Correlation coefficients shown in each box with p values below. (TIF) [file pone.0160192.s001.tif]

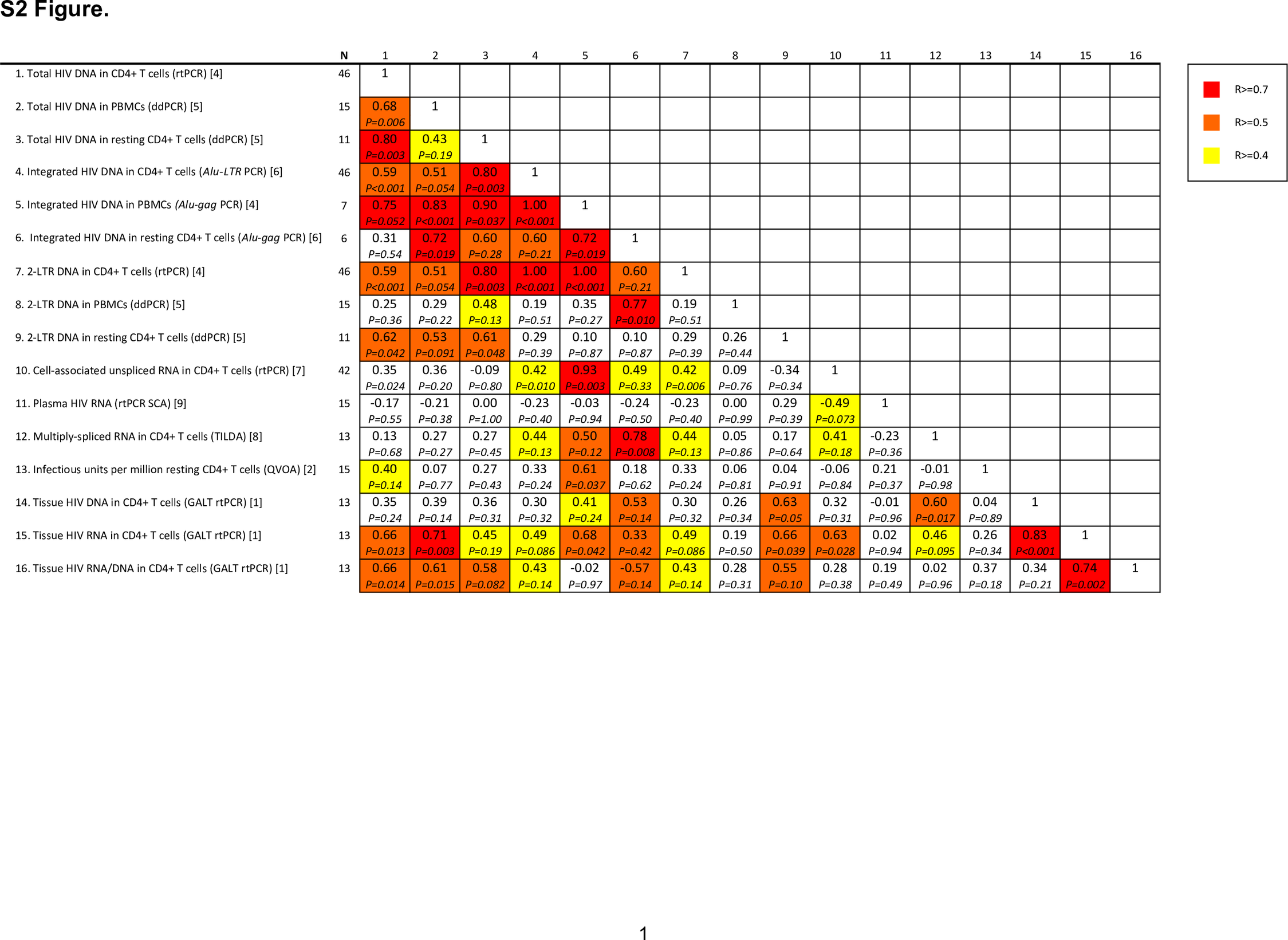

Supplement: S2 Fig — Abbreviations: rtPCR = reverse transcriptase polymerase chain reaction (PCR); ddPCR = droplet digital PCR; Alu PCR = PCR using a primer in an Alu element to detect integrated HIV-1 DNA; rCD4 = resting CD4+ T cells; PBMC = peripheral blood mononuclear cells; SCA = single copy assay. Correlation coefficients shown in each box with p values below. (TIF) [file pone.0160192.s002.tif]
