# Supplementary material for: Anti-HIV Antibody Responses and the HIV Reservoir Size during Antiretroviral Therapy
Source: PLoS One. 2016 Aug 2;11(8):e0160192. doi: 10.1371/journal.pone.0160192 (PMC4970722; doi:10.1371/journal.pone.0160192)
Supplement: S1 Table — (DOCX) [file pone.0160192.s003.docx]

**S1 Table.** Age-adjusted linear regressions of measures of the HIV reservoir and anti-HIV antibody responses^a^.

| **Total HIV-1 DNA** | | | | | | | | | | | | | | | |  |  |
| --- | --- | --- | --- | --- | --- | --- | --- | --- | --- | --- | --- | --- | --- | --- | --- | --- | --- |
|  | **rtPCR (CD4)** | | |  | | **ddPCR (PBMC)** | | |  | | **ddPCR (rCD4)** | | | |  |  |  |
|  | **(N = 46)** | | |  | | **(N = 20)** | | |  | | **(N = 11)** | | | |  |  |  |
| **Antibody** | **Fold-change^b^** | | | **P^c^** | | **Fold-change** | | | **P** | | **Fold-change** | | | | **P** |  |  |
| **GP120** | 1.02 (0.92, 1.12) | | | 0.75 | | 1.08 (0.96, 1.23) | | | 0.20 | | 1.07 (0.89, 1.28) | | | | 0.42 |  |  |
| **GP41** | 1.00 (0.96, 1.03) | | | 0.82 | | 1.03 (0.99, 1.07) | | | 0.092 | | 0.98 (0.94, 1.03) | | | | 0.34 |  |  |
| **RT** | 0.99 (0.93, 1.06) | | | 0.85 | | 1.05 (0.98, 1.12) | | | 0.17 | | 1.01 (0.91, 1.11) | | | | 0.85 |  |  |
| **INT** | 1.04 (0.92, 1.18) | | | 0.54 | | 1.15 (0.97, 1.38) | | | 0.10 | | 1.14 (0.95, 1.36) | | | | 0.14 |  |  |
| **PR** | 0.94 (0.78, 1.14) | | | 0.54 | | 1.34 (0.93, 1.93) | | | 0.11 | | 1.31 (0.87, 1.98) | | | | 0.16 |  |  |
| **MA** | 0.97 (0.81, 1.16) | | | 0.73 | | 0.89 (0.67, 1.20) | | | 0.43 | | 0.93 (0.67, 1.29) | | | | 0.63 |  |  |
| **CA** | 0.92 (0.80, 1.05) | | | 0.19 | | 0.89 (0.66, 1.21) | | | 0.44 | | 0.84 (0.61, 1.15) | | | | 0.24 |  |  |
| **Integrated HIV-1DNA** | | | | | | | | | | | | | | | |  |  |
|  | ***Alu*-*LTR* PCR (CD4)** | | |  | | ***Alu*-*LTR* PCR (PBMC)** | |  | | | ***Alu-gag* PCR (rCD4)** | | | |  |  |  |
|  | **(N = 46)** | | |  | | **(N = 12)** | |  | | | **(N = 10)** | | | |  |  |  |
| **Antibody** | **Fold-change^b^** | | | **P^c^** | | **Fold-change** | | **P** | | | **Fold-change** | | | | **P** |  |  |
| **GP120** | 1.07 (0.95, 1.19) | | | 0.25 | | 1.02 (0.75, 1.38) | | 0.91 | | | 0.87 (0.56, 1.33) | | | | 0.46 |  |  |
| **GP41** | 1.00 (0.96, 1.04) | | | 0.95 | | 1.00 (0.95, 1.06) | | 0.93 | | | 0.98 (0.91, 1.06) | | | | 0.55 |  |  |
| **RT** | 1.05 (0.97, 1.13) | | | 0.24 | | 1.04 (0.89, 1.21) | | 0.59 | | | 0.96 (0.77, 1.21) | | | | 0.71 |  |  |
| **INT** | 1.01 (0.87, 1.17) | | | 0.88 | | 1.02 (0.67, 1.54) | | 0.92 | | | 0.74 (0.39, 1.41) | | | | 0.31 |  |  |
| **PR** | 1.19 (0.95, 1.48) | | | 0.12 | | 1.51 (0.60, 3.78) | | 0.34 | | | 0.87 (0.21, 3.71) | | | | 0.83 |  |  |
| **MA** | 1.12 (0.91, 1.39) | | | 0.29 | | 0.67 (0.36, 1.25) | | 0.18 | | | **0.35 (0.18, 0.65)** | | | | **0.005** |  |  |
| **CA** | 1.03 (0.88, 1.21) | | | 0.69 | | 0.80 (0.44, 1.42) | | 0.40 | | | 0.5 (0.23, 1.11) | | | | 0.079 |  |  |
| **2-LTR HIV-1 DNA** | | | | | | | | | | | | | | |  |  |  |
|  | **rtPCR (CD4)** |  | | | **ddPCR (PBMC)** | |  | | | **ddPCR (rCD4)** | | |  | |  |  |  |
|  | **(N = 46)** |  | | | **(N = 20)** | |  | | | **(N = 11)** | | |  | |  |  |  |
| **Antibody** | **Fold-change^b^** | **P^c^** | | | **Fold-change** | | **P** | | | **Fold-change** | | | **P** | |  |  |  |
| **GP120** | 1.07 (0.95, 1.19) | 0.25 | | | 1.10 (0.91, 1.32) | | 0.32 | | | 1.12 (0.82, 1.52) | | | 0.44 | |  |  |  |
| **GP41** | 1.00 (0.95, 1.04) | 0.94 | | | 1.04 (0.99, 1.10) | | 0.12 | | | 0.99 (0.91, 1.07) | | | 0.77 | |  |  |  |
| **RT** | 1.04 (0.97, 1.12) | 0.25 | | | 1.07 (0.97, 1.18) | | 0.18 | | | 0.99 (0.84, 1.17) | | | 0.90 | |  |  |  |
| **INT** | 1.01 (0.87, 1.17) | 0.89 | | | 1.16 (0.89, 1.52) | | 0.26 | | | 1.17 (0.84, 1.63) | | | 0.29 | |  |  |  |
| **PR** | 1.18 (0.95, 1.48) | 0.13 | | | 1.52 (0.88, 2.59) | | 0.12 | | | 1.28 (0.59, 2.77) | | | 0.48 | |  |  |  |
| **MA** | 1.12 (0.90, 1.38) | 0.30 | | | 0.88 (0.57, 1.36) | | 0.55 | | | 1.02 (0.58, 1.81) | | | 0.92 | |  |  |  |
| **CA** | 1.03 (0.88, 1.21) | 0.70 | | | 1.02 (0.65, 1.61) | | 0.93 | | | 0.76 (0.44, 1.30) | | | 0.27 | |  |  |  |
| **HIV-1 RNA** | | | | | | | | | | | | | | |  |  |  |
|  | **CA-US RNA** |  | | | **Plasma RNA** | |  | | | **TILDA** | | |  | |  |  |  |
|  | **rtPCR (CD4)** |  | | | **rtPCR (plasma)** | |  | | | **msRNA (CD4)** | | |  | |  |  |  |
|  | **(N = 42)** |  | | | **(N = 20)** | |  | | | **(N = 18)** | | |  | |  |  |  |
| **Antibody** | **Fold-change^b^** | **P^c^** | | | **Fold-change** | | **P** | | | **Fold-change** | | | **P** | |  |  |  |
| **GP120** | **1.29 (1.07, 1.56)** | **0.009** | | | 0.84 (0.46, 1.56) | | 0.57 | | | 1.07 (0.84, 1.37) | | | 0.56 | |  |  |  |
| **GP41** | 1.04 (0.96, 1.13) | 0.33 | | | 0.89 (0.75, 1.05) | | 0.15 | | | 0.99 (0.93, 1.06) | | | 0.83 | |  |  |  |
| **RT** | 1.05 (0.92, 1.20) | 0.48 | | | 0.96 (0.69, 1.36) | | 0.82 | | | 1.00 (0.87, 1.15) | | | 1.00 | |  |  |  |
| **INT** | 1.11 (0.84, 1.46) | 0.46 | | | 0.95 (0.39, 2.33) | | 0.91 | | | 1.15 (0.81, 1.63) | | | 0.42 | |  |  |  |
| **PR** | 1.10 (0.73, 1.67) | 0.63 | | | 1.55 (0.24, 9.83) | | 0.62 | | | 1.37 (0.65, 2.89) | | | 0.39 | |  |  |  |
| **MA** | 1.15 (0.77, 1.70) | 0.49 | | | 1.27 (0.31, 5.18) | | 0.73 | | | 0.74 (0.42, 1.30) | | | 0.27 | |  |  |  |
| **CA** | 1.08 (0.81, 1.45) | 0.58 | | | 1.52 (0.36, 6.47) | | 0.55 | | | 0.78 (0.44, 1.39) | | | 0.38 | |  |  |  |
| **HIV-1 INFECTIOUS UNITS** | | | | |  |  |  |  |  |  |  |  |  |  |  |  |  |
|  | **QVOA** |  | | |  |  |  |  |  |  |  |  |  |  |  |  |  |
|  | **IUPM (rCD4)** |  | | |  |  |  |  |  |  |  |  |  |  |  |  |  |
|  | **(N = 20)** |  | | |  |  |  |  |  |  |  |  |  |  |  |  |  |
| **Antibody** | **Fold-change^b^** | **P** | | |  |  |  |  |  |  |  |  |  |  |  |  |  |
| **GP120** | 1.18 (0.89, 1.57) | 0.24 | | |  |  |  |  |  |  |  |  |  |  |  |  |  |
| **GP41** | 0.98 (0.9, 1.07) | 0.63 | | |  |  |  |  |  |  |  |  |  |  |  |  |  |
| **RT** | 1.03 (0.88, 1.22) | 0.67 | | |  |  |  |  |  |  |  |  |  |  |  |  |  |
| **INT** | 0.96 (0.63, 1.48) | 0.86 | | |  |  |  |  |  |  |  |  |  |  |  |  |  |
| **PR** | 1.64 (0.70, 3.86) | 0.24 | | |  |  |  |  |  |  |  |  |  |  |  |  |  |
| **MA** | 1.12 (0.57, 2.20) | 0.73 | | |  |  |  |  |  |  |  |  |  |  |  |  |  |
| **CA** | 0.78 (0.39, 1.56) | 0.47 | | |  |  |  |  |  |  |  |  |  |  |  |  |  |
| **HIV-1 DNA AND RNA IN TISSUE** | | | | | | | | | | | | | | | | | |
|  | **Total HIV-1 DNA** | |  | | **Total HIV-1 RNA^d^** | | |  | | | | **HIV-1 RNA/DNA** | |  | | |  |
|  | **rtPCR (CD4)** | |  | | **rtPCR (CD4)** | | |  | | | | **rtPCR (CD4)** | |  | | |  |
|  | (N = 16) | |  | | (N = 15) | | |  | | | | (N = 15) | |  | | |  |
| **Antibody** | **Fold-change^b^** | | **P^c^** | | **Fold-change** | | | **P** | | | | **Fold Change** | | **P** | | |  |
| **GP120** | 1.21 (1.01, 1.44) | | 0.040 | | 1.10 (1.00, 1.22) | | | 0.052 | | | | 1.71 (0.86, 3.41) | | 0.12 | | |  |
| **GP41** | 1.00 (0.94, 1.06) | | 0.94 | | 1.00 (0.97, 1.03) | | | 1.00 | | | | 1.08 (0.9, 1.29) | | 0.38 | | |  |
| **RT** | 1.05 (0.94, 1.17) | | 0.34 | | 1.02 (0.96, 1.09) | | | 0.53 | | | | 1.15 (0.77, 1.74) | | 0.46 | | |  |
| **INT** | **1.30 (1.03, 1.64)** | | **0.029** | | **1.17 (1.05, 1.30)** | | | **0.007** | | | | 2.33 (1.09, 4.98) | | 0.032 | | |  |
| **PR** | 1.84 (1.16, 2.91) | | 0.014 | | 1.33 (0.99, 1.78) | | | 0.054 | | | | 4.06 (0.54, 30.6) | | 0.16 | | |  |
| **MA** | 0.95 (0.64, 1.42) | | 0.80 | | 1.02 (0.81, 1.28) | | | 0.84 | | | | 2.38 (0.61, 9.34) | | 0.19 | | |  |
| **CA** | 0.90 (0.62, 1.31) | | 0.56 | | 0.95 (0.76, 1.18) | | | 0.60 | | | | 0.8 (0.2, 3.27) | | 0.74 | | |  |

Abbreviations: GP120 = envelope glycoprotein 120; GP41 = envelope glycoprotein 41; RT = reverse transcriptase; INT = integrase; PR = protease; MA = matrix; CA = capsid; rtPCR = reverse transcriptase polymerase chain reaction (PCR); LTR = long terminal repeat; Gag = HIV-1 Gag protein; ddPCR = droplet digital PCR; *Alu* PCR = PCR using a primer in an *Alu* element to detect integrated HIV-1 DNA; rCD4 = resting CD4+ T cells; PBMC = peripheral blood mononuclear cells. Associations with P<0.05 are highlighted in bold font.

^a^ Linear regressions of log_2_anti-HIV antibody levels adjusted for age. Note: outcome measures are shown in the rows in the left column while predictor variables are shown in columns.

^b^ Fold-change in anti-HIV antibody responses per fold-change in the HIV reservoir measure.

^c^ Bonferroni-adjusted significance cutoff would be P < 0.00045, after adjustment for 112 assessments of association.

^d^ Normalized to levels of glyceraldehyde phosphate dehydrogenase (GAPDH), as determined by a separate rtPCR.
