# Supplementary material for: Anti-HIV Antibody Responses and the HIV Reservoir Size during Antiretroviral Therapy
Source: PLoS One. 2016 Aug 2;11(8):e0160192. doi: 10.1371/journal.pone.0160192 (PMC4970722; doi:10.1371/journal.pone.0160192)
Supplement: S2 Table — (DOCX) [file pone.0160192.s004.docx]

**S2 Table.** Duration of ART-adjusted linear regressions of measures of the HIV reservoir and anti-HIV antibody responses^a^.

| **Total HIV-1 DNA** | | | | | | |
| --- | --- | --- | --- | --- | --- | --- |
|  | **rtPCR (CD4)** |  | **ddPCR (PBMC)** |  | **ddPCR (rCD4)** |  |
|  | **(N = 36)** |  | **(N = 16)** |  | **(N = 9)** |  |
| **Antibody** | **Fold-change^b^** | **P^c^** | **Fold-change** | **P** | **Fold-change** | **P** |
| **GP120** | 1.07 (0.95, 1.21) | 0.26 | 1.05 (0.9, 1.22) | 0.54 | 1.07 (0.87, 1.33) | 0.45 |
| **GP41** | 0.99 (0.95, 1.04) | 0.79 | 1.02 (0.98, 1.07) | 0.33 | 0.97 (0.92, 1.03) | 0.28 |
| **RT** | 1.01 (0.93, 1.10) | 0.76 | 1.06 (0.97, 1.15) | 0.18 | 1.02 (0.90, 1.15) | 0.74 |
| **INT** | 1.09 (0.93, 1.27) | 0.27 | 1.19 (0.96, 1.48) | 0.11 | 1.12 (0.89, 1.39) | 0.28 |
| **PR** | 1.00 (0.79, 1.25) | 0.98 | 1.37 (0.86, 2.18) | 0.17 | 1.34 (0.76, 2.37) | 0.25 |
| **MA** | 0.99 (0.78, 1.26) | 0.93 | 0.92 (0.66, 1.28) | 0.59 | 0.94 (0.71, 1.24) | 0.60 |
| **CA** | 0.93 (0.78, 1.10 | 0.36 | 0.83 (0.58, 1.18) | 0.27 | 0.79 (0.56, 1.11) | 0.14 |
| **Integrated HIV-1DNA** | | | | | | |
|  | ***Alu*-*LTR* PCR (CD4)** |  | ***Alu*-*LTR* PCR (PBMC)** |  | ***Alu-gag* PCR (rCD4)** |  |
|  | **(N = 36)** |  | **(N = 8)** |  | **(N = 7)** |  |
| **Antibody** | **Fold-change^b^** | **P^c^** | **Fold-change** | **P** | **Fold-change** | **P** |
| **GP120** | 1.08 (0.95, 1.21) | 0.23 | 1.19 (0.70, 2.04) | 0.44 | 0.96 (0.38, 2.44) | 0.91 |
| **GP41** | 1.00 (0.95, 1.04) | 0.89 | 1.02 (0.92, 1.12) | 0.66 | 1.01 (0.85, 1.2) | 0.87 |
| **RT** | 1.05 (0.96, 1.14) | 0.28 | 1.08 (0.80, 1.46) | 0.54 | 0.90 (0.55, 1.47) | 0.59 |
| **INT** | 1.01 (0.86, 1.18) | 0.88 | 0.95 (0.37, 2.43) | 0.90 | 0.52 (0.11, 2.39) | 0.30 |
| **PR** | 1.17 (0.94, 1.47) | 0.16 | 1.28 (0.17, 9.48) | 0.76 | 0.35 (0.02, 7.22) | 0.39 |
| **MA** | 1.13 (0.89, 1.43) | 0.30 | 0.85 (0.29, 2.50) | 0.71 | 0.36 (0.12, 1.08) | 0.06 |
| **CA** | 1.03 (0.87, 1.23) | 0.69 | 0.61 (0.19, 1.90) | 0.31 | 0.32 (0.06, 1.76) | 0.14 |

| **2-LTR HIV-1 DNA** | | | | | | | | | | |  |  |
| --- | --- | --- | --- | --- | --- | --- | --- | --- | --- | --- | --- | --- |
|  | **rtPCR (CD4)** |  | | **ddPCR (PBMC)** | |  | | **ddPCR (rCD4)** | |  |  |  |
|  | **(N = 36)** |  | | **(N = 16)** | |  | | **(N = 9)** | |  |  |  |
| **Antibody** | **Fold-change^b^** | **P^c^** | | **Fold-change** | | **P** | | **Fold-change** | | **P** |  |  |
| **GP120** | 1.07 (0.95, 1.21) | 0.24 | | 1.14 (0.92, 1.42) | | 0.20 | | 1.09 (0.71, 1.68) | | 0.65 |  |  |
| **GP41** | 1.00 (0.95, 1.04) | 0.88 | | 1.03 (0.97, 1.10) | | 0.28 | | 0.94 (0.85, 1.04) | | 0.19 |  |  |
| **RT** | 1.05 (0.96, 1.14) | 0.29 | | 1.05 (0.92, 1.20) | | 0.41 | | 0.99 (0.78, 1.27) | | 0.96 |  |  |
| **INT** | 1.01 (0.86, 1.18) | 0.89 | | 1.14 (0.8, 1.62) | | 0.45 | | 1.11 (0.69, 1.80) | | 0.60 |  |  |
| **PR** | 1.16 (0.93, 1.46) | 0.18 | | 1.52 (0.74, 3.09) | | 0.23 | | 1.44 (0.43, 4.81) | | 0.49 |  |  |
| **MA** | 1.13 (0.89, 1.43) | 0.32 | | 0.93 (0.57, 1.53) | | 0.76 | | 1.06 (0.61, 1.86) | | 0.80 |  |  |
| **CA** | 1.03 (0.87, 1.23) | 0.70 | | 0.98 (0.56, 1.72) | | 0.93 | | 0.59 (0.32, 1.10) | | 0.084 |  |  |
| **HIV-1 RNA** | | | | | | | | | | |  |  |
|  | **CA-US RNA** |  | | **Plasma RNA** | |  | | **TILDA** | |  |  |  |
|  | **rtPCR (CD4)** |  | | **rtPCR (plasma)** | |  | | **msRNA (CD4)** | |  |  |  |
|  | **(N = 33)** |  | | **(N = 16)** | |  | | **(N = 15)** | |  |  |  |
| **Antibody** | **Fold-change^b^** | **P^c^** | | **Fold-change** | | **P** | | **Fold-change** | | **P** |  |  |
| **GP120** | 1.23 (1.00, 1.52) | 0.053 | | 0.79 (0.39, 1.59) | | 0.48 | | 1.23 (0.89, 1.69) | | 0.19 |  |  |
| **GP41** | 1.03 (0.94, 1.12) | 0.52 | | 0.81 (0.69, 0.96) | | 0.02 | | 1.03 (0.95, 1.12) | | 0.49 |  |  |
| **RT** | 1.04 (0.90, 1.21) | 0.56 | | 0.85 (0.57, 1.26) | | 0.39 | | 1.04 (0.85, 1.27) | | 0.71 |  |  |
| **INT** | 1.08 (0.81, 1.44) | 0.61 | | 0.79 (0.26, 2.40) | | 0.66 | | 1.13 (0.66, 1.95) | | 0.62 |  |  |
| **PR** | 1.09 (0.71, 1.68) | 0.68 | | 0.95 (0.09, 9.67) | | 0.96 | | 1.60 (0.53, 4.83) | | 0.37 |  |  |
| **MA** | 1.02 (0.65, 1.60) | 0.93 | | 1.01 (0.22, 4.68) | | 0.99 | | 0.75 (0.36, 1.56) | | 0.41 |  |  |
| **CA** | 1.04 (0.76, 1.44) | 0.78 | | 1.39 (0.25, 7.76) | | 0.68 | | 0.57 (0.26, 1.23) | | 0.14 |  |  |
| **HIV-1 INFECTIOUS UNITS** | | | |  |  |  |  |  |  |  |  |  |
|  | **QVOA** |  | |  |  |  |  |  |  |  |  |  |
|  | **IUPM (rCD4)** |  | |  |  |  |  |  |  |  |  |  |
|  | **(N = 16)** |  | |  |  |  |  |  |  |  |  |  |
| **Antibody** | **Fold-change^b^** | **P** | |  |  |  |  |  |  |  |  |  |
| **GP120** | 1.10 (0.79, 1.52) | 0.55 | |  |  |  |  |  |  |  |  |  |
| **GP41** | 0.96 (0.87, 1.05) | 0.34 | |  |  |  |  |  |  |  |  |  |
| **RT** | 1.04 (0.86, 1.25) | 0.70 | |  |  |  |  |  |  |  |  |  |
| **INT** | 0.91 (0.54, 1.51) | 0.68 | |  |  |  |  |  |  |  |  |  |
| **PR** | 1.53 (0.54, 4.31) | 0.39 | |  |  |  |  |  |  |  |  |  |
| **MA** | 0.98 (0.48, 1.98) | 0.95 | |  |  |  |  |  |  |  |  |  |
| **CA** | 0.66 (0.31, 1.40) | 0.25 | |  |  |  |  |  |  |  |  |  |
| **HIV-1 DNA AND RNA IN TISSUE** | | | | | | | | | | | | |
|  | **Total HIV-1 DNA** | |  | | **Total HIV-1 RNA^d^** | |  | | **HIV-1 RNA/DNA** | | |  |
|  | **rtPCR (CD4)** | |  | | **rtPCR (CD4)** | |  | | **rtPCR (CD4)** | | |  |
|  | (N = 12) | |  | | (N = 11) | |  | | (N = 11) | | |  |
| **Antibody** | **Fold-change^b^** | | **P^c^** | | **Fold-change** | | **P** | | **Fold Change** | | | **P** |
| **GP120** | 1.32 (1.06, 1.65) | | 0.018 | | 1.17 (1.02, 1.33) | | 0.027 | | 2.00 (0.72, 5.53) | | | 0.16 |
| **GP41** | 1.00 (0.92, 1.09) | | 0.95 | | 1.00 (0.96, 1.05) | | 0.93 | | 1.00 (0.75, 1.34) | | | 0.98 |
| **RT** | 1.11 (0.96, 1.30) | | 0.14 | | 1.05 (0.96, 1.15) | | 0.23 | | 1.26 (0.70, 2.29) | | | 0.40 |
| **INT** | 1.27 (0.88, 1.84) | | 0.18 | | 1.17 (0.99, 1.37) | | 0.064 | | 1.79 (0.52, 6.14) | | | 0.31 |
| **PR** | 2.08 (1.01, 4.28) | | 0.049 | | 1.39 (0.88, 2.20) | | 0.14 | | 3.66 (0.14, 92.7) | | | 0.38 |
| **MA** | 1.06 (0.69, 1.64) | | 0.76 | | 1.03 (0.8, 1.33) | | 0.80 | | 2.35 (0.53, 10.41) | | | 0.22 |
| **CA** | 0.78 (0.46, 1.33) | | 0.32 | | 0.82 (0.61, 1.10) | | 0.15 | | 0.28 (0.04, 1.87) | | | 0.16 |

Abbreviations: GP120 = envelope glycoprotein 120; GP41 = envelope glycoprotein 41; RT = reverse transcriptase; INT = integrase; PR = protease; MA = matrix; CA = capsid; rtPCR = reverse transcriptase polymerase chain reaction (PCR); LTR = long terminal repeat; Gag = HIV-1 Gag protein; ddPCR = droplet digital PCR; *Alu* PCR = PCR using a primer in an *Alu* element to detect integrated HIV-1 DNA; rCD4 = resting CD4+ T cells; PBMC = peripheral blood mononuclear cells. Associations with P<0.05 are highlighted in bold font.

^a^ Linear regressions of log_2_anti-HIV antibody levels adjusted for age. Note: outcome measures are shown in the rows in the left column while predictor variables are shown in columns.

^b^ Fold-change in anti-HIV antibody responses per fold-change in the HIV reservoir measure.

^c^ Bonferroni-adjusted significance cutoff would be P < 0.00045, after adjustment for 112 assessments of association.

^d^ Normalized to levels of glyceraldehyde phosphate dehydrogenase (GAPDH), as determined by a separate rtPCR.
